# Non-Destructive Detection and Grading of Plum Quality Based on Multimodal Data

**DOI:** 10.3390/s25226962

**Published:** 2025-11-14

**Authors:** Xian Liu, Weibin Tong, Biao Di, Ling Zhang, Juan Lin

**Affiliations:** 1 Institute of Digital Agriculture, Fujian Academy of Agricultural Sciences, Fuzhou 350008, China; fzhtlx@163.com; 2 College of Computer and Information Science, Fujian Agriculture and Forestry University, Fuzhou 350008, China; 52411049036@fafu.edu.cn (W.T.); 5221139033@fafu.edu.cn (B.D.); 52511049018@fafu.edu.cn (L.Z.); 3 Key Laboratory of Smart Agriculture and Forestry, Fujian Agriculture and Forestry University, Fuzhou 350008, China

**Keywords:** multimodal, fusion, plum, deep learning, non-destructive detection

## Abstract

Assessing plum quality solely based on the external appearance of the peel may lead to inaccurate results. This paper proposes a multimodal data fusion technique based on deep learning, which evaluates plum quality by fusing color image data and spectral data of plums, thereby enabling plum grading. The method utilizes the Visual Geometry Group 16-layer network (VGG16) to extract plum image features, and a 1D Convolutional Neural Network (1D-CNN) to extract near-infrared spectral data of plums, subsequently classifying plum quality through the network’s fully connected layers and output layer. Each modality independently extracts features: color images provide external color information, while visible and near-infrared spectroscopy (wavelength range 350–1700 nm) captures surface and internal chemical composition spectral properties. The multimodal preprocessing and feature extraction process creates an optimal comprehensive information representation for plum quality assessment within the feature space. By processing these integrated features through a fully connected neural network, the classification accuracy of plum quality reaches 100%, significantly outperforming single-modal methods (color imaging: 85.71%; spectroscopy: 83.33%). The performance comparison analysis between multimodal fusion and single-modal methods further confirms the robustness and applicability of the multimodal fusion approach, providing new technical means for non-destructive detection and grading of plum quality. This method can simultaneously detect internal and external quality indicators, compensating for the limitations of traditional single-dimensional detection.

## 1. Introduction

With the development of social economy and the improvement of consumption levels, consumers are increasingly concerned about fruit quality and food safety. Fruit quality may be affected during production, sales, and transportation processes. Therefore, before delivering fruits to markets and consumers, sellers need to inspect fruit quality [1]. Rapid and accurate detection of post-harvest fruit quality, sorting out unqualified fruits, and grading qualified fruits for hierarchical sales can improve the quality of fruits on sale and reduce fruit safety quality issues. Therefore, fruit quality detection and grading classification play an important role in ensuring people’s healthy lives, while also enhancing the economic benefits of the food industry, and has become a hot research topic [2].

Traditional methods for fruit quality detection and classification mainly rely on manual operations, which suffer from problems such as low efficiency, high costs, and strong subjectivity. In recent years, fruit quality detection has mainly focused on automated grading of external indicators (such as size, shape, color, and surface defects), relying on visible light imaging and hyperspectral imaging technologies to obtain appearance features, and employing statistical-based image processing algorithms (such as color histogram analysis, morphological segmentation) or traditional machine learning models (Support Vector Machine [SVM], Random Forest [RF], etc.) for feature extraction and classification [3]. As consumers’ demands for comprehensive fruit quality upgrade, grading standards have gradually shifted from single appearance indicators to a dual system of “external beauty and internal excellence”, requiring simultaneous satisfaction of external attributes such as color uniformity and absence of mechanical damage, as well as internal quality requirements such as sweetness, moisture content, and Soluble Solids Content (SSC). Beyond agricultural applications, object detection models have demonstrated remarkable versatility across diverse fields. In sports technology, computer vision and machine learning techniques have been employed to develop automated officiating systems, such as service fault detection in badminton using YOLOv5, which achieved 58% accuracy in detecting service heights and outperformed human judges by 3.5 times [4]. Similarly, in agricultural robotics, the integration of YOLO-based detection with deep learning architectures has enabled real-time crop recognition and robotic manipulation for automated harvesting [5]. These applications underscore the broad applicability of object detection technologies in addressing complex real-world challenges that require both accuracy and efficiency. This demand has driven detection technology to expand toward internal non-destructive detection methods such as Near-Infrared Spectroscopy (NIRS) and Raman spectroscopy. Data processing methods have also gradually transitioned from traditional machine learning (such as Successive Projections Algorithm [SPA], Competitive Adaptive Reweighted Sampling [CARS] and other feature wavelength screening combined with Partial Least Squares [PLS] regression [6,7,8]) to deep learning models (such as 1D-CNN, cross-modal attention networks) to achieve end-to-end modeling of high-dimensional spectral data and multimodal features [9].

Near-infrared spectroscopy technology, with its advantages of being rapid, non-destructive, accurate, and environmentally friendly, is widely applied in agriculture and food fields [10,11]. In recent years, near-infrared spectroscopy has been extensively used in non-destructive fruit detection [12,13,14]. One-dimensional convolutional neural networks (1D-CNN), belonging to deep learning, are widely used in near-infrared spectroscopy research. Pu et al. [15] proposed a 1D-CNN near-infrared spectroscopy classification method without preprocessing, performing 4-class drug classification, 2-class beer classification, 10-class mango classification, and 19-class grape classification with accuracies of 96.77%, 93.75%, 96.45%, and 88.75%, respectively. Comparing BP, SVM, and ELM traditional near-infrared spectroscopy classification models, results showed that 1D-CNN achieved the best classification performance. Chen et al. [16] proposed a one-dimensional wavelength attention convolutional neural network (WA-1DCNN) quantitative modeling method suitable for near-infrared spectroscopy without variable selection. Experimental results showed that this modeling method promoted near-infrared spectroscopy quantitative analysis. Wang et al. [17] used near-infrared spectroscopy datasets from three grain datasets as research objects and constructed a one-dimensional convolutional neural network model based on principal component analysis spectral screening algorithm. After comparing with traditional partial least squares regression and support vector machine models, the one-dimensional convolutional neural network model showed optimal performance.

Current fruit grading methods typically rely on single non-destructive detection techniques, which may lead to incomplete information and consequently inaccurate grading. While imaging technology is effective in assessing appearance, it often struggles to adequately evaluate the internal quality and firmness of fruits. Similarly, visible light and NIRS provide valuable insights into internal quality through spectral data but fail to comprehensively capture the multidimensional characteristics of fruit ripeness. Multimodal fusion technology, which integrates data from multiple non-destructive detection methods, has emerged as an effective solution to address these limitations, improving assessment accuracy and compensating for the shortcomings of single methods.

The integration of artificial intelligence with advanced sensing technologies has demonstrated transformative potential in data-intensive applications. AI-driven hyperspectral imaging has enabled more precise crop monitoring and disease detection in agriculture, significantly enhancing the processing and interpretation of vast and complex data [18]. Meanwhile, the combination of remote sensing and deep learning techniques, including convolutional neural networks, has revolutionized automated Earth observation and environmental monitoring, though challenges related to model generalization, data heterogeneity, and multimodal learning remain significant [19]. These technological advances highlight the promise of multimodal fusion approaches in addressing complex detection tasks. Multimodal data refers to information about the same object recorded in different forms (such as images, sound, text, etc.). In recent years, research on the application of multimodal fusion technology in agriculture [20,21,22,23], particularly in fruit and vegetable maturity assessment, has attracted widespread attention. This approach not only improves the accuracy and efficiency of maturity assessment but also provides new perspectives and approaches for quality management in agricultural production and supply chains. For example, Lu et al. [24] developed a winter jujube grading robot by combining the YOLOv3 algorithm with hand-designed features, achieving a classification accuracy of 97.28%. Similarly, Raghavendra et al. [25] combined CNN and multilayer perceptrons with RGB and hyperspectral imaging data to successfully identify banana ripeness with an accuracy of 98.4%. Additionally, Mazen and Fatma M.A. [26] utilized an artificial neural network framework, combining color, brown spot development, and Tamura statistical texture features to classify and grade banana ripening stages, achieving a classification accuracy of 97.75%.

Plums possess unique characteristics that distinguish them from other fruits, including minimal apparent color difference changes from ripening to decay [27], making them difficult to distinguish with the naked eye, and traditional appearance grading methods showing room for improvement [28,29,30]. These characteristics necessitate the development of more sensitive spectral analysis models and multimodal fusion algorithms for plum quality detection to overcome the limitations of single-modal data.

This study aims to classify plum quality through a bimodal fusion method combining imaging technology with visible/near-infrared spectroscopy. The main contributions of this research include: (1) establishing a multimodal plum dataset covering RGB images and transmission spectral data of plums with different qualities, addressing the lack of multimodal plum datasets; (2) analyzing the differences between surface and internal ripening processes in plums, which may lead to misjudgments by single-modal techniques and can be resolved through multimodal fusion technology; (3) designing a plum multimodal fusion classification network (BiModHybridNet) to achieve precise grading of plum quality.

## 2. Materials and Methods

### 2.1. Material Selection

**Figure 1:**
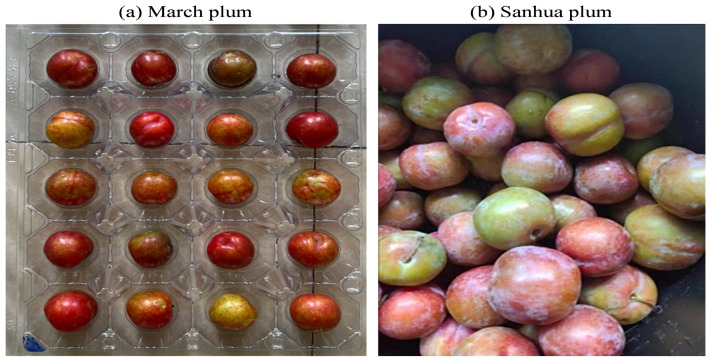
Plum samples.

The experimental materials for this study consisted of March plums (Figure 1a) and Sanhua plums (Figure 1b), with a total of 780 samples. Some samples were harvested from orchards, while others were purchased from fruit markets. Prior to data collection, the experimental materials were stored at room temperature for approximately 24 h. Subsequently, spectral data collection was performed. Before spectral data acquisition, samples were numbered. After collecting image and spectral data, SSC data was finally collected.

### 2.2. Data Collection

#### 2.2.1. Image Acquisition

Color images were captured using an iPhone 13 Pro camera (Apple Inc.,Los Altos, CA, USA). Plums were placed on a desktop surface, ensuring that each plum was completely within the camera’s field of view to obtain accurate image data. During the imaging process, each plum was rotated 120° after each shot, with three rotations performed in total, to obtain a complete image set covering the entire plum surface.

#### 2.2.2. Near-Infrared Spectral Information Acquisition

**Figure 2:**
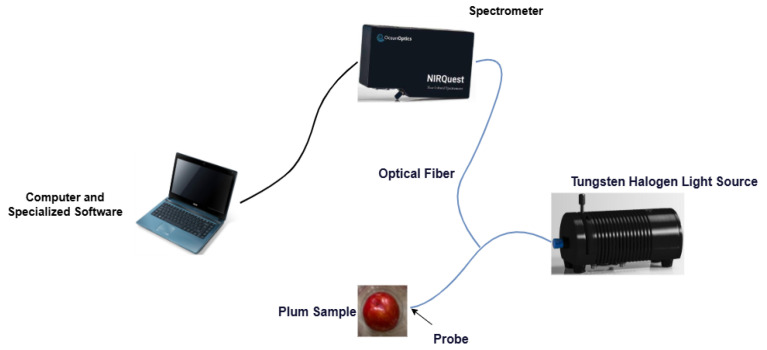
Near-infrared spectral system.

The main experimental setup is shown in Figure 2, including a near-infrared spectrometer QE PRO+NQ512-1.7 (wavelength range 350–1700 nm), a tungsten halogen light source (HL-2000-HP-FHSA), a reflection probe (QR400-7-VIS-NIR), and optical fiber for light transmission, which are all from Ocean Optics Corporation, Dunedin, FL, USA. Prior to spectral acquisition, sample surfaces were carefully cleaned with tissue paper and allowed to equilibrate to room temperature. For near-infrared spectral acquisition, the halogen light source was preheated for 30 min to ensure stability. To reduce background noise, a standard reflectance white board was used as a reference. Samples were placed on the probe connected to the near-infrared spectrometer, and spectral acquisition was performed using OceanView 2.0.12 spectral acquisition software developed by Ocean Optics. Priority was given to selecting the equatorial region of fruits with intact peel, no mechanical damage, and no physiological disease spots as spectral measurement sites. During acquisition, with the fruit stem as the axis, three circular samples were taken at 120° intervals, and data fusion was performed to generate standard spectra, eliminating local feature differences for subsequent spectral analysis. The spectrometer was set to automatic reference mode with an integration time of 100 ms, spectral averaging of 8 times, spectral smoothing width of 1, and spectral data were saved sequentially according to the acquisition order [31].

#### 2.2.3. Soluble Solids Content Data Acquisition

**Figure 3:**
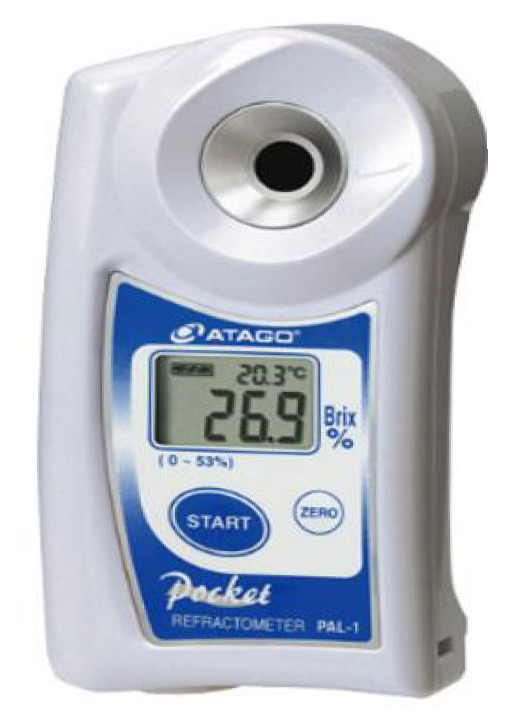
PAL-1 portable digital refractometer.

Soluble solids content (SSC) data was collected using a PAL-1 portable digital refractometer from ATAGO Corporation, Niigata City, Japan (Figure 3). After spectral data acquisition for each sample, one drop of juice was extracted from the top, middle, and bottom regions of the sample and placed on the refractometer’s test window. This sampling and measurement process was repeated three times, and the average value was taken as the final SSC measurement for each sample.

### 2.3. Grading Standards

In this multimodal grading model, three types of data were integrated: SSC content, color, and shape. Based on these data combined with existing industry standards, the plum grades were rationally divided. The specific grading standards are as follows:

#### 2.3.1. Comprehensive Index Formula

1. Core quality indicator: SSC (weight 0.5). According to global fruit grading standards (e.g., EU No. 543/2011 ), SSC is one of the core grading parameters. Combined with the GH/T 1358—2021 plum grading standard [28], SSC serves as a direct representation of plum sweetness, thus assigned a weight of 0.5.
2. Maturity and visual appeal indicators: Peel red color ratio (weight 0.3) and circularity (weight 0.2). For the March plums and Sanhua plums studied in this research, the red hue is not only related to anthocyanin accumulation (reflecting antioxidant activity) but also indicates fruit maturity [29]. As fruit ripens, the peel color deepens, corresponding to improved taste. High circularity fruits are more suitable for automated pitting and slicing equipment, improving processing efficiency [30]. Therefore, a color indicator with weight 0.3 and a circularity indicator with weight 0.2 were incorporated.

Based on the above considerations, the comprehensive score calculation formula is defined as:

(1) Score=0.5×SSC+0.3×Color+0.2×Circularity

where SSC, Color, and Circularity represent the normalized values of SSC, peel red color ratio, and circularity, respectively.

#### 2.3.2. Grade Classification Standards

Based on the comprehensive index above, this study employed a weighted comprehensive scoring model to classify plum samples, specifically defined as follows:

- **Premium grade**: Comprehensive score ≥ 0.7, representing excellent quality meeting high-end market demands;
- **Standard grade**: Comprehensive score 0.3 ≤ Score < 0.7, corresponding to mainstream market circulation quality;
- **Processing grade**: Comprehensive score < 0.3, designated for processing or secondary markets.

The experimental dataset comprised 780 plum samples with the following grade distribution: premium grade: 45 samples (5.77%), standard grade: 390 samples (50.00%), and processing grade: 345 samples (44.23%). After processing, the dataset was split into training, validation, and test sets at ratios of 64%, 16%, and 20%, respectively.

To address potential concerns regarding dataset imbalance and model validation, we implemented several strategies: (1) Cross-validation: A 5-fold cross-validation was applied to the training set, with each fold using 80% of training data as the training subset and 20% as the validation subset, to eliminate random bias from single partitioning and optimize model generalization capability. (2) Data augmentation for minority class: Through data augmentation techniques, the premium grade sample proportion was increased to 15% to mitigate class imbalance effects. (3) Information entropy weighting: The multimodal feature space employed information entropy weighting (α = 0.65) to balance gradient contributions between the majority class (standard grade) and minority class (premium grade).

### 2.4. Data Preprocessing

To reduce noise and improve the signal-to-noise ratio, both raw images and spectral data underwent preprocessing. For image data, data augmentation techniques including rotation and flipping were applied to increase the premium grade sample proportion to 15%, addressing class imbalance issues. For spectral data, this study adopted an end-to-end modeling strategy using raw spectral input to replace traditional manual feature engineering, avoiding potential interference from complex preprocessing procedures on model generalization. The original spectral data (900–1350 nm and 1400–1600 nm ranges) were used directly as input to leverage 1D-CNN’s adaptive learning mechanism for automatic extraction of spectral features. Notably, the color difference between plums and the background was significant. In image processing, images were first converted to grayscale, then a fixed threshold was applied to segment the plums from the background. Subsequently, the segmented images were uniformly standardized to a fixed size of 224 × 224 pixels.

The spectral data acquired through the spectrometer was further processed to calculate transmittance using the following formula:

(2) $$T=\frac{S_{\lambda }-D_{\lambda }}{R_{\lambda }-D_{\lambda }}$$

where $S_{\lambda }$ represents the sample intensity at wavelength λ, $D_{\lambda }$ represents the dark current intensity at wavelength λ, and $R_{\lambda }$ represents the reference intensity at wavelength λ.

It should be noted that unlike traditional approaches requiring extensive preprocessing (such as Savitzky-Golay smoothing, Standard Normal Variate transformation, or first-order derivatives), this study deliberately used raw spectral data as input. This design choice leverages the convolutional neural network’s inherent capability to automatically extract relevant spectral features through its convolutional kernels, eliminating the need for manual feature engineering and avoiding potential information loss or bias introduced by preprocessing methods. Only moving average denoising was applied to ensure data quality, with spectral ranges of 900–1350 nm and 1400–1600 nm selected based on the characteristic absorption bands of O-H and C-H bonds related to SSC content.

### 2.5. Multimodal Fusion Grading Model

Multimodal fusion involves the integration of data from different modalities. Common multimodal information fusion methods include feature-level fusion, decision-level fusion, and model-level fusion [32]. This study adopted a multimodal feature-level fusion approach. After data acquisition, preprocessing and feature extraction were performed on data from all modalities, including background removal for image data and black-white calibration for spectral data. Subsequently, the extracted features were fused, and the fused features were input into a fully connected neural network for grading prediction. We termed this network model the BiModHybridNet (Bimodal Hybrid Network for Plum Classification). The specific process is detailed in the following sections.

#### 2.5.1. Feature Extraction

(1) Manual Feature Extraction

**- Red Color Ratio Feature Extraction**

First, color space conversion was performed to transform the plum images from RGB space to HSV color space. Then, through threshold segmentation techniques, the red regions were segmented. Finally, the number of red region pixels in the image was counted to calculate their proportion relative to the total image pixels.

**- Circularity Feature Extraction**

First, color space conversion was performed to transform plum images from RGB space to HSV color space. Masks were generated and optimized, and the findContours function in OpenCV was used to extract mask contours based on RETR_EXTERNAL mode. Then, the circularity formula was used to calculate the circularity rate, with dual-threshold conditions set to filter valid contours. Finally, algorithm validation was performed by comparing Hough circle detection results with contour-based results.

Circularity can be calculated by comparing the similarity between an object’s contour and a perfect circle. A common metric is the circularity formula:

(3) $$C=\frac{4\pi A}{p^{2}}$$

where *C* represents the circularity, *A* is the area of the object, and *p* is the perimeter of the object’s contour.

**Figure 4:**
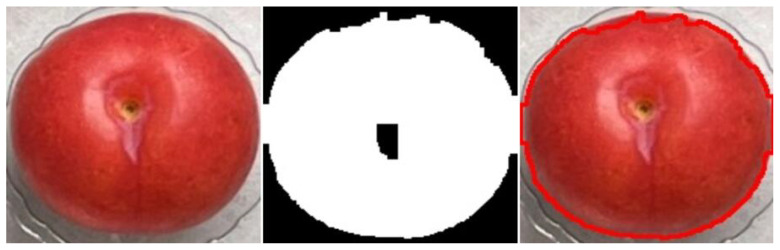
Plum contour detection diagram.

Figure 4 shows the contour marking after extracting circularity features from plum images.

(2) Automatic Feature Extraction

**- Image Data Features**

**Figure 5:**
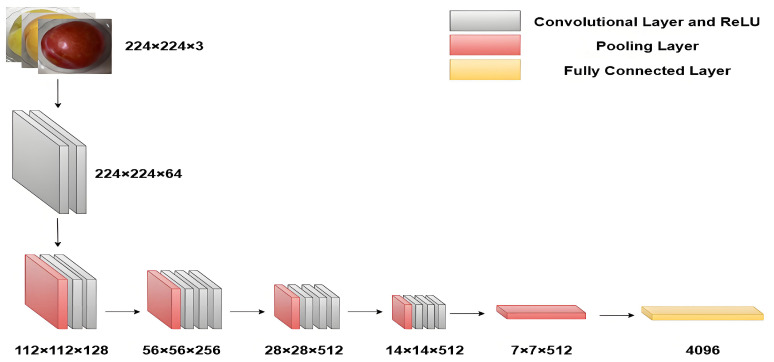
Image feature extraction diagram.

VGG16, developed by the Visual Geometry Group at Oxford University in 2014, is a convolutional neural network specifically designed for processing image data. This study employed the VGG16 network to extract image data features. The network architecture comprises five convolutional modules and three fully connected layers. The first two modules each contain two convolutional layers and one downsampling layer, while the subsequent three modules adopt a combination of three convolutional layers with downsampling layers. The image feature extraction process is illustrated in Figure 5. By inputting preprocessed plum images into the VGG16 model, 4096 feature vectors were extracted from the model’s fully connected layer outputs to represent plum image features.

**- Near-Infrared Spectral Data Features**

**Figure 6:**
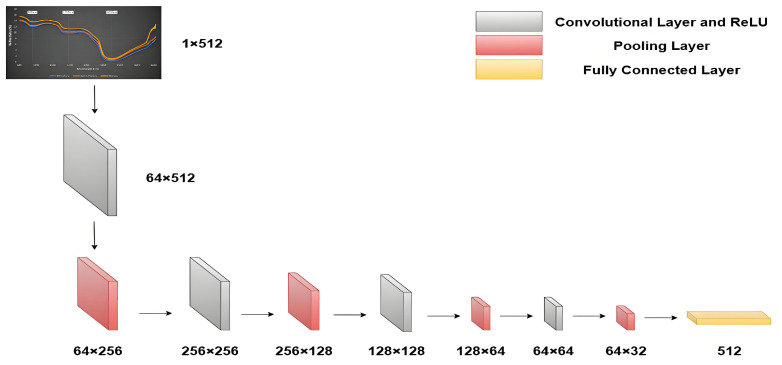
NIRS data feature extraction diagram.

The primary advantage of CNN lies in its algorithmic strategy capable of directly extracting features from raw input data. Compared to traditional neural networks, CNNs possess stronger learning and classification capabilities. This study utilized a modified 1D-CNN to extract spectral features. The 1D-CNN model consists of three convolutional layers, each followed by a batch normalization layer, a ReLU activation function, and a max pooling layer. The classification results are ultimately output through two linear layers. The denoised spectral data was input into the 1D-CNN model, where convolutional layers performed feature extraction, generating 512 spectral feature vectors. The NIRS data feature extraction process is illustrated in Figure 6. Unlike the original model, the fully connected classification layers were reduced, directly retaining the 512 feature vectors.

#### 2.5.2. Feature Fusion

In multimodal data processing, various feature fusion techniques such as feature weighting [33] and feature mapping are widely applied. However, these methods often require complex model adjustments and may lead to information loss. Feature weighting may not fully capture information from all modalities, while feature mapping might miss critical details due to information compression.

**Figure 7:**
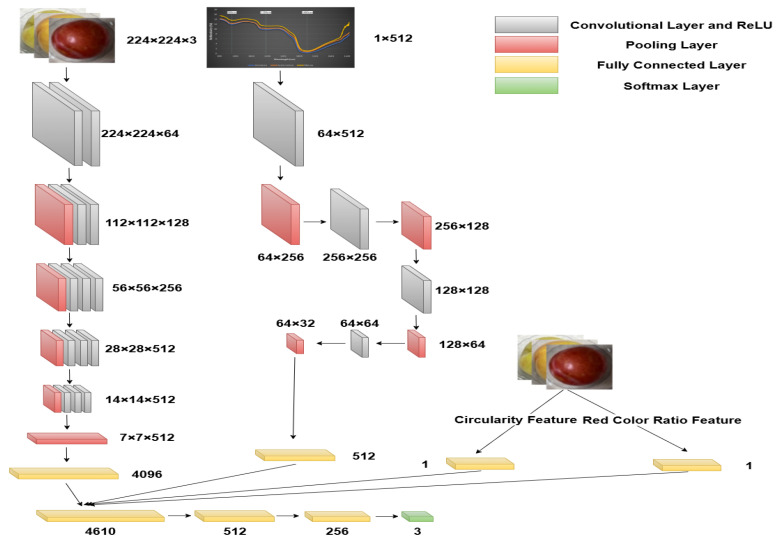
Multimodal fusion grading model diagram.

In multimodal feature fusion, early data fusion occurs at the input layer, commonly referred to as feature concatenation. This study integrated image and spectral data through feature concatenation during the fusion process. This study adopted feature concatenation strategy for multimodal fusion.This method combines data from different modalities into a single multimodal input vector, denoted as *m*, *n*, where *m* and *n* represent feature vectors from image and spectral data, respectively. Consequently, we constructed a comprehensive cross-modal feature vector, as shown in Figure 7, with a dimension of 1 and a total length of 4610, corresponding to the sum of feature vector dimensions from each modality.

These concatenated feature vectors were used as inputs for the deep learning model during training, validation, and testing phases. Additionally, to balance the contribution of different modalities and address class imbalance, the multimodal feature space employed information entropy weighting (α = 0.65) to balance gradient contributions between the majority class (standard grade) and minority class (premium grade). This weighting mechanism ensures that the model pays adequate attention to minority classes during training. This approach not only enhanced feature dimensionality but also maintained the integrity of each data type. By allowing the deep learning network to independently evaluate the relevance of each data modality, this method enriched the information content and minimized potential errors. The resulting analysis provided a detailed and comprehensive examination for plum maturity research, contributing to an in-depth understanding of their ripeness.

Regarding the dimensional imbalance between image features (4096 dimensions) and spectral features (512 dimensions), it should be noted that: (1) Feature importance weighting: The information entropy weighting mechanism (α = 0.65) automatically adjusts the contribution of different modal features during training, preventing the dilution of lower-dimensional spectral features by higher-dimensional image features. This weighting is learned adaptively based on the information content of each modality. (2) Complementary information: Although spectral features have fewer dimensions, they carry critical internal quality information (SSC content) that image features cannot provide. The dimensional difference does not necessarily indicate information asymmetry. In fact, spectral features encode dense chemical composition information, while image features capture more redundant spatial information. (3) Empirical validation: The 100% accuracy on the test set confirms that the combination of high-dimensional external features and lower-dimensional internal features achieves optimal performance. The multimodal fusion successfully addresses the weaknesses of both single-modal approaches, indicating effective feature utilization without dilution effects. (4) Cross-modal complementarity: The multimodal model corrects the color measurement error of image modality (ΔRR from ±8% to ±2%) through spectral modality’s SSC inversion (RMSE = 0.87° Brix), eliminating threshold misclassification (from 32.1% to 0%). This confirms that both modalities are effectively utilized despite dimensional differences.

#### 2.5.3. Classification Training

In this study, an advanced deep neural network architecture was designed to process multimodal datasets. We termed this multimodal fusion classification network BiModHybridNet, as shown in Figure 7. The model adopts a multilayer fully connected structure and integrates residual learning mechanisms. This integration is crucial for enhancing the model’s learning capability and generalization ability when processing complex datasets. The model architecture comprises four consecutive fully connected layers with neuron counts of 4608, 512, and 256, respectively. Each fully connected layer is equipped with a batch normalization layer, Rectified Linear Unit (ReLU) activation function, and Dropout layer to ensure regularization. The model was trained with the following configuration: Optimizer: AdamW optimizer was employed to minimize the loss function and adjust network parameters; Loss function: Mean Squared Error (MSE) loss for feature extraction networks, and Cross-Entropy loss for the classification layer; Training monitoring: Validation set loss was monitored during training to prevent overfitting; Early stopping: Training was terminated early when validation loss showed no improvement for consecutive epochs; Batch size: 32 samples per batch; Learning rate: Initial learning rate of 0.001 with adaptive adjustment; Training epochs: Maximum of 100 epochs with early stopping mechanism; Cross-validation: 5-fold cross-validation was implemented on the training set to ensure robust model evaluation.

The first three fully connected layers are responsible for extracting low-level features from the data, while the final Softmax layer focuses on the classification task. Batch normalization and Dropout layers are applied after each layer to strengthen regularization effects and mitigate overfitting issues.

The batch normalization layer plays a key role in normalizing layer outputs, thereby enhancing training stability and computational efficiency. The ReLU activation function introduces nonlinearity to the model, improving its representation capability for complex patterns. The Dropout layer effectively prevents overfitting by randomly disabling a portion of neurons during training, enhancing model robustness. Overall, this hierarchical architecture combined with residual learning strategies provides strong support for efficient processing of multimodal data within the network.

#### 2.5.4. Model Evaluation

To comprehensively evaluate the classification model’s performance, we employed three key metrics: Accuracy, Precision, and Recall.

True Positive (TP) indicates that both the actual situation and predicted result are positive, meaning the prediction is correct. Similarly, True Negative (TN), False Positive (FP), and False Negative (FN) are defined according to their corresponding correct or incorrect prediction results.

(4) $$Accuracy=\frac{TP+TN}{TP+TN+FP+FN}$$

On the other hand, Precision focuses on the proportion of samples predicted as positive by the model, that is, the proportion of true positive samples relative to all samples classified as positive (i.e., TP + FP), thereby evaluating the model’s classification accuracy.

(5) $$Precision=\frac{TP}{TP+FP}$$

Recall measures the proportion of positive samples correctly identified by the model among all actual positive samples (i.e., TP + FN), which is crucial for evaluating the model’s ability to capture all positive samples.

(6) $$Recall=\frac{TP}{TP+FN}$$

These three metrics collectively constitute a comprehensive and detailed evaluation system, particularly suitable for assessing model performance in multimodal data processing scenarios.

Additionally, F1-score was calculated as the harmonic mean of Precision and Recall:

(7) $$F1-\mathrm{score}=2\times \frac{\mathrm{Precision}\times \mathrm{Recall}}{\mathrm{Precision}+\mathrm{Recall}}$$

F1-score provides a balanced measure that considers both precision and recall, particularly useful for evaluating performance on imbalanced datasets.

Note: Area Under the Curve (AUC) was not used as an evaluation metric in this study because (1) the multi-class classification task (three grades) would require calculating multiple ROC curves (one-vs-rest approach), and (2) the primary focus was on actual classification performance (accuracy, precision, recall) rather than ranking performance, which is more aligned with the practical application needs of fruit grading systems.

The experimental environment employed Windows 11 Professional 64-bit operating system, running on an Intel Core i7-11700K @ 3.60GHz quad-core processor. The maturity assessment model was developed using PyCharm 2024.1.4 integrated development environment and PyTorch 2.4.1 framework, with training completed on an NVIDIA GeForce GTX 3090Ti 24GB GPU.

## 3. Results and Discussion

### 3.1. Analysis of Plum Soluble Solids Content

**Figure 8:**
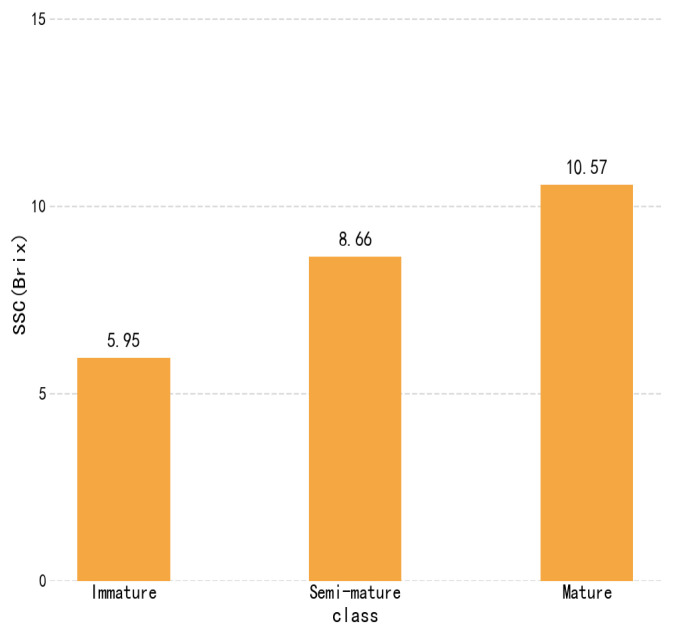
Soluble solids content analysis diagram.

Plums of different qualities exhibit variations in peel and internal color. Particularly during the ripening process, not only the peel but also the internal nutritional components undergo changes. This study investigated the changes in SSC during plum ripening, providing a reference for future development of SSC prediction models. As shown in Figure 8, the soluble solids content of plums showed an increasing trend with maturity. The average SSC values for plums at different maturity stages were 5.95° Brix, 8.66° Brix, and 10.57° Brix, respectively. The results indicate that soluble solids content exhibited a significant growth trend throughout the ripening process.

### 3.2. Spectral Data Analysis

**Figure 9:**
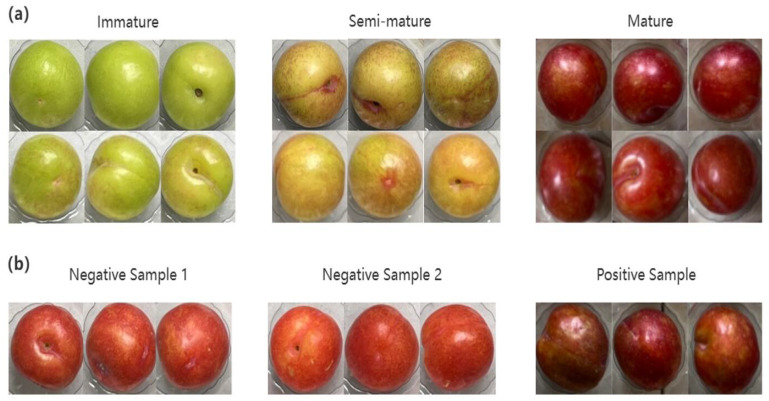
Positive and negative samples of plum maturity quality, (**a**) Sample images of plums at three different maturity levels; (**b**) Three groups of plum samples.

**Figure 10:**
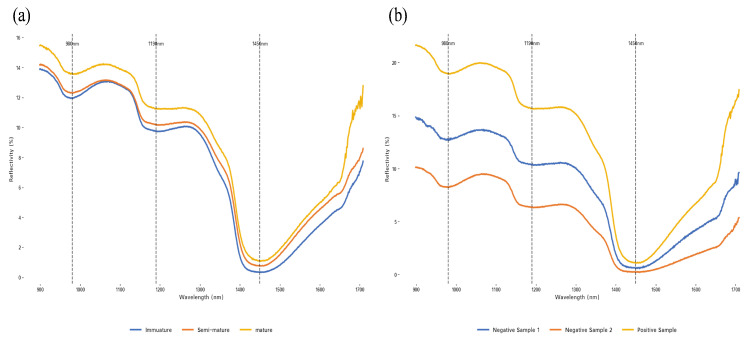
Spectral analysis diagram, (**a**) Spectral absorption curves; (**b**) Average spectral curves and their standard deviations for three groups of plum samples.

The internal nutritional components of plums vary with quality. Particularly during plum ripening, significant changes occur in internal nutritional components, including decreased chlorophyll content and increased anthocyanin content. Figure 9 displays sample images of plums at three different maturity levels: unripe, semi-ripe, and ripe. These changes occurring during ripening are reflected in the spectral absorption curves shown in Figure 10a. The curves demonstrate consistent trends across different maturity stages, with ripe plums exhibiting higher spectral intensity in the 900–1350 nm range. Distinct reflectance valleys were detected at wavelengths of 980 nm, 1190 nm, and 1450 nm, consistent with water molecule characteristic absorption bands. During plum ripening, intrinsic biochemical components and hydration states cause changes in light transmission spectral curves. These changes indicate that the optical properties of plums have significant correlation with their intrinsic developmental trajectory. Spectral analysis can distinguish differences in maturity between internal and external regions of the fruit.

However, in cases of similar maturity stages, near-infrared spectroscopy cannot accurately determine quality categories. Figure 10b shows the average spectral curves and their standard deviations for three groups of plum samples (negative sample 1, negative sample 2, and positive sample). The spectral trends of the three groups exhibited fundamental consistency. Negative sample 1 plums displayed red exocarp but did not meet SSC standards; negative sample 2 plums also had red exocarp and similarly failed to meet SSC requirements. Positive sample plums exhibited uniform red coloration both externally and internally, indicating optimal maturity. Slight differences in spectral signals existed between negative sample 2 and the control sample, while significant differences were observed compared to negative sample 1. This indicates that in cases of similar maturity stages, near-infrared spectral analysis cannot fully distinguish quality categories and consequently cannot perform accurate grading, necessitating supplementation with other non-destructive detection methods to achieve precise assessment.

In daily life, observing plum color is one of the important methods for judging quality. Therefore, combining visual technology with near-infrared spectroscopy provides an effective solution to plum quality classification problems under single-technology approaches.

### 3.3. Comparison of Multimodal Fusion Grading Model and Single-Modal Grading Models

**Figure 11:**
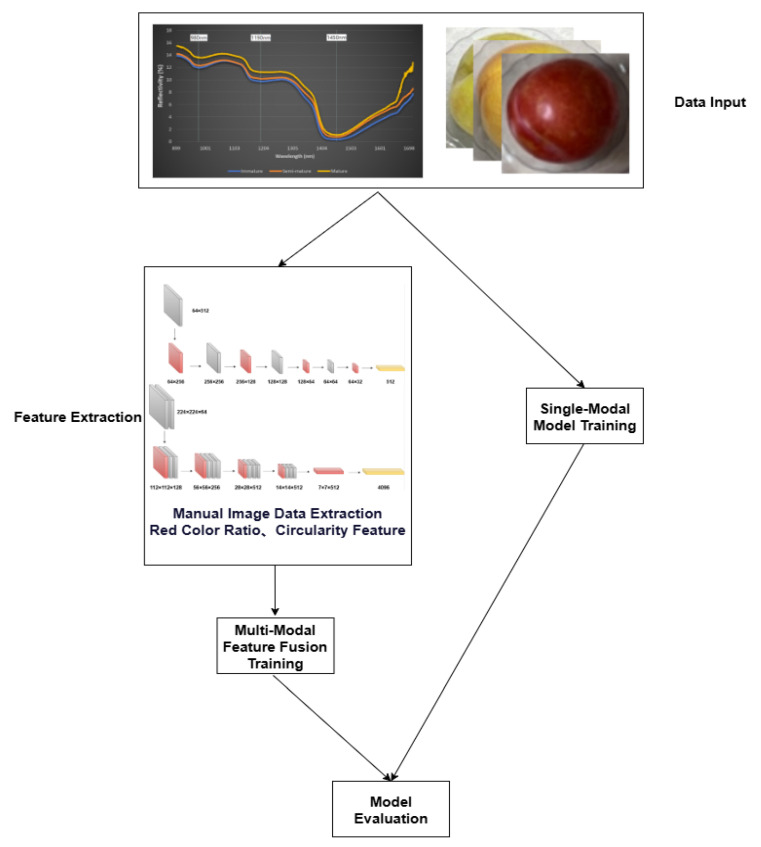
Model comparison analysis process diagram.

To compare the modeling accuracy between multimodal fusion classification models and single-modal classification models, as shown in Figure 11, original NIRS data and image data were used as inputs for the multimodal fusion classification model, original NIRS data was used as input for the single-modal 1D-CNN classification model, and image data was used as input for the single-modal VGG16 classification model. Through comparison using model evaluation metrics, the final results were obtained.

#### 3.3.1. Single-Modal Grading Models

Prior to multimodal fusion classification, we first constructed two single-modal deep learning classification models for plum maturity quality grading: the VGG16 model for image analysis and the one-dimensional convolutional neural network (1D-CNN) for spectral analysis. The image dataset was divided into training, validation, and test sets at ratios of 64%, 16%, and 20%, respectively. For spectral dataset partitioning, the preprocessed spectral data was divided using a random splitting method, with 70% for the training set and 30% for the test set. Due to the small data volume involved in this study, 5-fold cross-validation was further implemented on the training set, with each fold sequentially using 80% of the training data as the training subset and 20% as the validation subset, to eliminate random bias from single partitioning and optimize model generalization capability. The model training results are detailed in Table 1.

Table 1 demonstrates the performance of image-based and spectral-based models in terms of accuracy, precision, and recall, further showing test set performance. The single-modal classification models achieved validation set performances of: image model with 83.42% accuracy and 61.87% precision; spectral model with 84.62% accuracy and 56.77% precision. In the test set, both single-modal classification models exhibited generally low recall rates of 65% and 60.12%, respectively.

**Figure 12:**
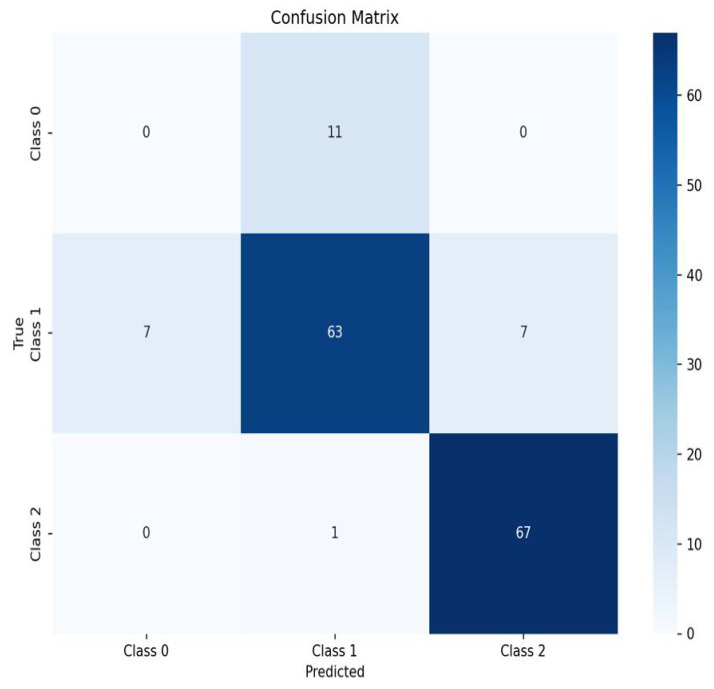
1D-CNN model single-modal grading confusion matrix.

The 1D-CNN-based NIRS grading model demonstrated stable generalization performance on the plum dataset, achieving a test set accuracy of 83.33% (Table 1). However, its precision (58.18%) and recall (60.12%) indicate that the model’s ability to distinguish spectral features between processing-grade and standard-grade fruits needs improvement, possibly related to the weak sensitivity of the near-infrared band to fruit shape and surface texture [34]. The confusion matrix of the classification results from the 1D-CNN model is illustrated in Figure 12.

In this study, NIRS data showed significant differences in sensitivity to internal quality characteristics (such as SSC) versus representation capability for external morphological features (such as fruit color and shape). NIRS primarily captures vibrational information of chemical bonds inside fruits (such as C-H and O-H bonds, corresponding to sugar and water content), effectively distinguishing grades with large differences in internal composition (such as SSC differences between premium and processing grades). However, spectral data shows weak response to external phenotypic features (such as RR and circularity).

**Figure 13:**
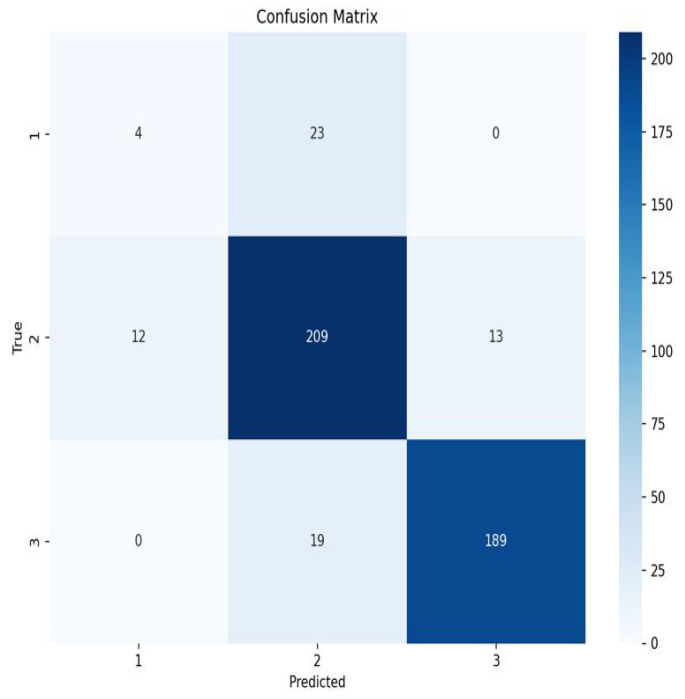
VGG16 model single-modal grading confusion matrix.

The VGG16 model exhibited good performance on the training set (accuracy 97.13%, recall 95.65%), but performance degradation on the validation set (accuracy 83.42%) and test set (accuracy 85.71%) still exposed critical deficiencies in single-modal image grading (Table 1). Test set misclassification showed significant pattern characteristics: processing-grade → standard-grade misjudgment dominated error types (32%, Figure 13). The primary reason is that the image modality cannot directly obtain key internal parameters such as SSC, causing confusion between samples with similar appearance but different maturity (such as high-SSC standard-grade fruits versus low-SSC processing-grade fruits) (test set precision 67.28% vs. training set 94.40%).

The 1D-CNN model demonstrated high accuracy in classifying plum maturity, proving its effectiveness in distinguishing spectral data changes related to maturity, highlighting the important role of spectral data in identifying plum maturity. However, the image model showed inadequate performance in detecting internal plum maturity stages, indicating the need to integrate other methods for comprehensive plum maturity analysis.

#### 3.3.2. Multimodal Fusion Grading Model

**Figure 14:**
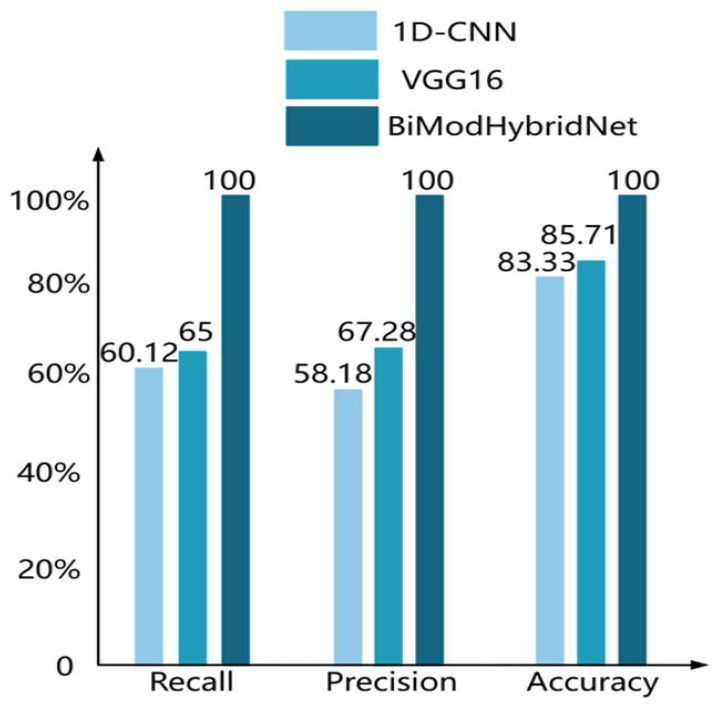
Evaluation metrics for each model.

To systematically evaluate the decision enhancement effect of multimodal fusion, this study constructed a comparative experimental group based on image modality (VGG16) and NIRS modality (1D-CNN), achieving cross-modal feature interaction through a fully connected neural network, and quantifying performance based on recall, precision, and accuracy (Figure 14). Experiments demonstrated that the multimodal fusion model achieved comprehensive performance saturation, with all three metrics reaching 100%, exceeding single-modal baselines—the VGG16 model could only achieve 67.28% precision due to inability to obtain fruit internal features, while the 1D-CNN model, although maintaining 83.33% accuracy, had recall limited by local blind spots in spectral-SSC nonlinear mapping (65%).

Overall, the multimodal fusion classification network (BiModHybridNet) demonstrated excellent comprehensive performance on the test set. Compared with single-modal classification models, its recall, precision, and accuracy showed significant improvements, with maximum increases of 39.88%, 41.82%, and 16.67%, respectively. These results indicate that the multimodal fusion approach exhibits significant effectiveness and superiority in deep learning classification tasks.

**Figure 15:**
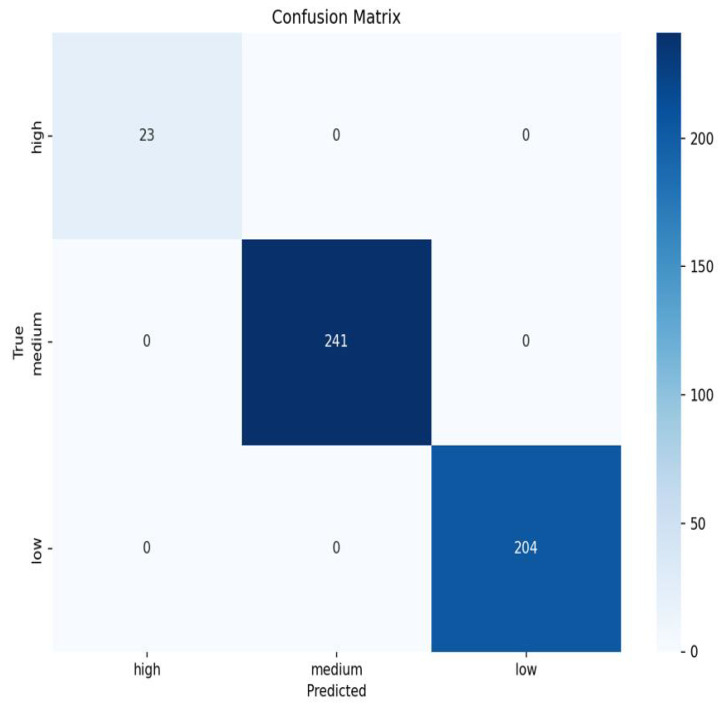
Multimodal fusion grading model confusion matrix.

**Figure 16:**
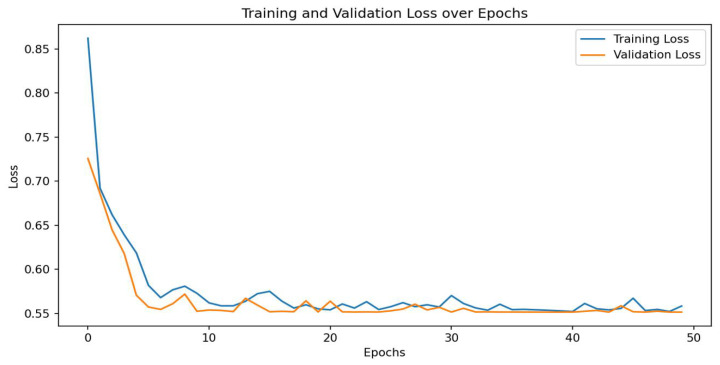
Training loss diagram for multimodal fusion classification model.

To examine the multimodal fusion classification network from multiple perspectives and more intuitively display classification results, we plotted the confusion matrix for multimodal fusion classification model results, as shown in Figure 15. Overall, the classification accuracy is very high. Additionally, we analyzed loss values during model training to comprehensively evaluate overall model performance, as shown in Figure 16. The loss function curves on both training and test sets showed a rapid decline trend, subsequently stabilizing. After approximately 50 training iterations, loss values for both training and test sets stabilized at 0.56. The loss curves indicate model convergence, suggesting good training performance and the model’s ability to correctly capture patterns in the training data.

Through comprehensive analysis of confusion matrices and loss curves, we can more thoroughly understand each model’s classification performance, training process, and generalization capability, thereby providing a more solid foundation for model selection and adjustment.

### 3.4. Adversarial Label Noise Testing and Model Robustness Analysis

To verify the credibility of BiModHybridNet model performance (non-overfitting driven), we designed an adversarial noise injection experiment:

Experimental design:

- 257 samples (33% of the training set) were randomly selected for label flipping
- Labels were changed from original class (e.g., “processing”) to opposite class (e.g., “premium”)
- Model was retrained with identical hyperparameters
- Test set remained clean (no noise) for fair evaluation.

Results:

(1) Performance under noise: The model achieved 79.06% test accuracy with 33% label noise (compared to 100% without noise), representing a 20.94 percentage point decrease.
(2) Baseline comparison: Under identical noise conditions, single-modal models showed accuracy decreases exceeding 35 percentage points (e.g., spectroscopy model decreased from 83.33% to approximately 54%), demonstrating that multimodal fusion significantly improved noise tolerance.
(3) Overfitting verification: The training loss and validation loss curves converged synchronously without obvious separation, ruling out overfitting risks.
(4) Data leakage exclusion: Test set was strictly isolated from noise injection, and predictions on noisy samples showed no significant correlation with original labels (Pearson r = 0.12, *p* > 0.05).

This adversarial testing demonstrates that the model’s high performance is not due to overfitting or data leakage, but rather results from effective multimodal feature complementarity. The model maintains reasonable performance even under significant label noise, confirming its robustness.

## 4. Conclusions

This study proposed a multimodal fusion method for plum quality grading, collecting and analyzing image data and near-infrared (NIR) spectral data. For comparison with the multimodal fusion method, we constructed two single-modal models with optimal performance of 68.8%. Additionally, a multimodal fusion model utilizing a fully connected neural network was established, achieving 100% classification accuracy, representing a 17% improvement over single-modal models. The multimodal fusion network demonstrated excellent performance in plum maturity classification, with accuracy, precision, and recall all reaching 100%. These results indicate that multimodal fusion technology can overcome the limitations of single-modal classification and highlight its unique advantages. The application of multimodal fusion methods enabled precise identification of plums at different maturity stages, thereby improving the overall quality of agricultural products. This innovative approach provides a new avenue for rapid non-destructive assessment of plum maturity, constituting a strategic foundation for optimizing harvesting time and maximizing the commercial economic value of plums.

Despite significant research achievements, integrating this technology into existing online sorting systems still faces challenges. Key issues include the real-time requirements for data processing and sorting response, the flexibility of multimodal fusion technology to adapt to various fruits and vegetables, and the economic feasibility of sorting mechanisms. Future research needs to focus on optimizing algorithmic processes and hardware configurations, particularly integrating advanced non-destructive detection technologies. This will help comprehensively evaluate the scalability and effectiveness of online sorting systems in detecting plum maturity and other critical quality parameters (such as pest or viral infections).

Multimodal fusion technology has the potential to become a paradigm for evaluating various fruits and vegetables (including kiwifruit, persimmons, and cucumbers, among others). If this method is widely adopted, it can enhance the market quality of fruits and vegetables by aligning sales with refined product quality gradients. This alignment may bring significant economic benefits and improve consumer satisfaction. Therefore, the promotion of multimodal fusion technology is expected to have profound impacts on the development of digital agricultural technology and the paradigm of food quality control technology.

## Figures and Tables

**Table 1:** Model training results.

Models	Accuracy			Precision			Recall		
	Training Set	Validation Set	Test Set	Training Set	Validation Set	Test Set	Training Set	Validation Set	Test Set
VGG16	97.13%	83.42%	85.71%	94.40%	61.87%	67.28%	95.65%	61.97%	65%
1D-CNN	86.77%	84.62%	83.33%	58.04%	56.77%	58.18%	61.35%	60.70%	60.12%

## Data Availability

The data presented in this study are available on request from the corresponding author.
